# Treatment of Urinary Tract Infections in Neonates and Young Infants: Evidence and Recommendations

**DOI:** 10.3390/antibiotics15080788

**Published:** 2026-08-14

**Authors:** Manar O. Lashkar, Milap C. Nahata

**Affiliations:** 1 Department of Pharmacy Practice, Faculty of Pharmacy, King Abdulaziz University, Jeddah 22254, Saudi Arabia; mlashkar@kau.edu.sa; 2 Institute of Therapeutic Innovations and Outcomes, College of Pharmacy, The Ohio State University, 500 West 12th Ave., Columbus, OH 43210, USA; 3 College of Medicine, The Ohio State University, Columbus, OH 43210, USA

**Keywords:** urinary tract infection, neonate, infant, antibiotic treatment, stewardship, bacteremia, neonatal pharmacokinetics

## Abstract

**Background/Objectives:** Urinary tract infection (UTI) is among the most common serious bacterial infections occurring in the first 90 days of life and a leading indication for parenteral antibiotic therapy. Despite its clinical importance, no randomized trial has compared empiric antibiotic regimens, route of administration, or treatment duration specifically in this population. This review synthesizes current evidence to support rational antibiotic treatment decisions for neonates (ages 0–28 days) and young infants (ages 29–90 days), including those born prematurely. **Methods:** A targeted PubMed and MEDLINE search was conducted through July 2026. Studies reporting treatment outcomes, pharmacokinetic data, or antibiotic safety in infants aged 0–90 days were included. Broader pediatric data were incorporated where age-specific evidence was unavailable and are presented with explicit population caveats. **Results:** *Escherichia coli* is the predominant uropathogen across all age strata; extended-spectrum beta-lactamase (ESBL)-producing organisms account for a clinically important minority of community-acquired isolates. No comparative trial of empiric regimens existed for confirmed UTI in this age group. Shorter intravenous courses were not associated with higher 30-day recurrence rates for either nonbacteremic or bacteremic UTI after exclusion of meningitis. Intravenous-to-oral (IV-to-oral) transition with amoxicillin–clavulanate was pharmacokinetically supported and clinically safe in selected term neonates, based on indirect evidence from neonatal infection trials. Non-carbapenem therapy is appropriate for ESBL UTI without concomitant bacteremia in clinically improving infants. Exclusion of meningitis before shortening intravenous therapy or initiating oral step-down is essential, particularly in neonates ≤ 28 days of age. **Conclusions:** Short parenteral courses with timely transition to oral antibiotics are recommended in appropriately selected infants with UTI, consistent with antimicrobial stewardship principles and supported by observational evidence. The evidence base is limited, with key gaps in empiric regimen selection, management of premature infants, ESBL treatment in bacteremic patients, and optimal duration of parenteral and oral antibiotic therapy.

## 1. Introduction

Urinary tract infection is among the most common serious bacterial infections found in the first 90 days of life, yet its management in this age group remains poorly standardized and inadequately studied. Unlike older children, neonates and young infants frequently present without obvious localized symptoms. Fever may be absent, and prolonged jaundice, poor feeding, lethargy, or unexplained clinical deterioration may be the only manifestations [1]. This diagnostic ambiguity means antibiotic therapy is often initiated empirically before UTI is confirmed.

UTI is the leading serious bacterial infection in febrile infants in the first months of life. In a multicenter secondary analysis of 3825 infants age ≤ 60 days, UTI was the most common serious bacterial infection, occurring in 7.6% of afebrile and 11% of febrile infants presenting to the emergency department [2]. In a vaccinated European cohort of children aged 0–36 months with fever without a source, all identified serious bacterial infections were urinary tract infections, with absence of occult bacteremia [3]. These findings emphasize UTI as the dominant infection requiring consideration and treatment in this age group.

A key feature that distinguishes UTI in neonates and young infants from older children is the risk of concurrent invasive bacterial infection. Among febrile infants aged ≤ 90 days with a positive urinalysis in the Febrile Infants Diagnostic Assessment and Outcome (FIDO) prospective cohort, bacteremia was present in 7.1% and bacterial meningitis in 0.9%; no case of meningitis occurred in infants over 60 days of age or in those with confirmed UTI [4]. These findings have direct implications for the selection of antibiotics and their route of administration, treatment duration, and monitoring, and underpin the need to exclude meningitis in deciding the treatment approach described in Section 7.3.

Despite the predominance and potential severity of UTI in this age group, no randomized trial has directly compared empiric antibiotic regimens, route of administration, or total treatment duration in culture-confirmed UTI in infants aged 0–90 days. Current practice is driven by observational data, extrapolation from non-UTI neonatal infection trials, expert consensus, and data from older children—each carrying important limitations that this review aims to make explicit.

Two systematic reviews have addressed the duration of intravenous antibiotic therapy for UTI specifically in this age group. Hikmat and colleagues reviewed 18 studies totaling 16,615 infants ≤ 90 days and found that shorter intravenous courses (≤7 days for bacteremic, and ≤3 days for nonbacteremic UTI) were not associated with higher recurrence rates [5]. A living systematic review by Nama and colleagues in infants < 2 months confirmed this finding and additionally found no significant difference in 30-day recurrence with ≤10 versus >10 total days of therapy [6]. However, both reviews were restricted to the question of antibiotic duration, with searched literature ending in early 2021. Neither review addressed empiric antibiotic selection, oral dosing based on pharmacokinetics, management of resistant organisms, treatment of UTI in premature infants, complicated UTI, meningitis-exclusion implications, or treatment failure. A short commentary examined treatment duration and oral antibiotic therapy in newborns [7]. To our knowledge, no comprehensive, treatment-focused synthesis covering the full antibiotic management pathway from empiric antibiotic selection through definitive antibiotic therapy, treatment of resistant organisms, and antibiotic stewardship has been published for this population. This review addresses those gaps, incorporating the most up-to-date evidence.

This review focuses on the therapeutic management of UTI in neonates (age 0–28 days) and young infants (29–90 days), including premature infants (gestational age < 37 weeks). It is directed at multidisciplinary clinicians and antimicrobial stewardship teams involved in the care of neonates and young infants. Diagnosis, urine collection methods, imaging, antimicrobial prophylaxis, and febrile-infant risk-stratification algorithms are addressed where they directly influence an antibiotic treatment decision.

## 2. Methods

A targeted literature search of PubMed and MEDLINE was conducted through July 2026. Search terms included: urinary tract infection, neonate, young infant, antibiotic treatment, empiric therapy, intravenous antibiotics, oral antibiotics, intravenous-to-oral transition, duration of therapy, bacteremia, ESBL, extended-spectrum beta-lactamase, antimicrobial resistance, pharmacokinetics, and neonatal. Boolean operators were applied to combine terms. English-language studies reporting treatment outcomes, pharmacokinetic data, or antibiotic safety in infants aged 0–90 days were included. Randomized controlled trials, systematic reviews, meta-analyses, observational cohort studies, and clinical practice guidelines were included. Case reports were included only where they illustrated a clinically important presentation or pathogen. Conference abstracts and non-peer-reviewed sources were excluded.

Where evidence specific to infants 0–90 days was unavailable, broader pediatric data (including infants up to 6 months or 1 year of age) and indirect evidence from non-UTI neonatal infection trials were incorporated. Each such source is presented with an explicit population caveat, and the evidence tier is specified throughout: *direct evidence* (studies reporting treatment outcomes specifically in 0–90-day old infants with UTI); *broader infant population* (studies including infants outside this range where results were not age-separable); *indirect evidence* (trials or studies in sepsis, possible serious bacterial infection, or other infection types extrapolated to UTI); and *expert practice* (where no direct or indirect evidence existed and guidance derived from pharmacological reasoning, guideline consensus, or clinical principle). Recommendations are labeled accordingly throughout, and no recommendation is stated more strongly than justified with its evidence.

Three age strata are used throughout: premature infants (gestational age < 37 weeks); neonates (postnatal age 0–28 days); and young infants (postnatal age 29–90 days). Where sources used different age boundaries, those are stated explicitly.

Studies were excluded if they did not report antibiotic treatment outcomes, pharmacokinetic data, or antibiotic safety specifically in infants aged 0–90 days, or if they addressed exclusively diagnostic, imaging, prophylaxis, or febrile-infant risk-stratification outcomes without reference to therapeutic management decisions. Formal GRADE methodology was not applied: the evidence base consisted predominantly of retrospective observational studies and indirect randomized controlled trial (RCT) data derived from non-UTI indications, which did not support the certainty-of-evidence scoring that GRADE requires at its higher levels. The explicit evidence-tiering system used throughout—direct, broader infant population, indirect, and expert practice—provides a transparent alternative that calibrates the strength of each recommendation to its supporting evidence. No formal registration of this narrative review was performed.

A narrative review was selected because the evidence base for this population is predominantly observational, heterogeneous in design, and insufficient in volume to support the pooled quantitative synthesis that a systematic review requires. Direct evidence from randomized controlled trials in infants aged 0–90 days with confirmed UTI is lacking; the synthesis therefore integrates indirect trial data, pharmacokinetic models, and observational cohort studies that are not amenable to formal meta-analytic pooling. The principal limitation of this approach is the potential for selection bias in source inclusion. To minimize this, search terms and inclusion criteria were prespecified, scope was bounded to antibiotic treatment outcomes only, and every evidence statement is labeled by its tier and population source.

## 3. Microbiology and Resistance Patterns Relevant to Empiric Therapy

Knowledge of the predominant uropathogens and their resistance profiles is the foundation of rational empiric antibiotic selection. Because resistance rates vary substantially by geographic setting, healthcare antibiotic exposure, and institutional context, local antibiogram data must be the primary guide for empiric antibiotic regimen selection. No data from the published literature can substitute for this. The following summary draws on studies that include infants in the 0–90-day age range, supplemented by broader pediatric UTI data where age-specific evidence was unavailable.

### 3.1. Predominant Uropathogens

*Escherichia coli* (*E. coli*) is the predominant uropathogen across all age strata. In a multicenter retrospective cohort of 115 infants aged ≤ 60 days with bacteremic UTI, *E. coli* accounted for 81% of causative organisms; the remaining isolates included *Enterococcus faecalis* (5%), *Staphylococcus* aureus (4%, all associated with urinary tract anomalies), *Klebsiella* spp. (3%), *Streptococcus* agalactiae (3%), *Enterobacter* spp. (3%), and *Citrobacter* spp. (1%) [8]. The presence of *Enterococcus faecalis* and Group B *Streptococcus* in this age group underscores the importance of coverage with empiric antibiotic therapy that extends beyond Gram-negatives alone in neonates.

A single-center cohort of 403 infants < 60 days of age (median age 21 days, IQR 8–39) provided UTI-specific uropathogen data in this age range: *E. coli* 60%, *Enterococcus faecalis* 13%, *Klebsiella* spp. 8%, *Enterobacter* spp. 8%, *Pseudomonas aeruginosa* 2%, and *Staphylococcus* species 2% [9]. A study restricted to term neonates ≤ 28 days (n = 112, 2 academic hospitals) identified *E. coli* in 66%, *Enterococcus* spp. in 9%, *K. pneumoniae* in 6%, Group B *Streptococcus* in 4%, and *S. aureus* in 4%; all isolates were susceptible to at least one available oral antibiotic [10]. Empiric regimens in another cohort were ampicillin plus cefotaxime (38%) and ampicillin plus gentamicin or tobramycin (33%) the first direct UTI-specific empiric regimen data in this age group [9]. In a single-center cohort of 451 infants aged ≤ 90 days, *E. coli* susceptibility was 50% to ampicillin, 100% to amikacin, 100% to meropenem, and 93% to gentamicin [11], confirming the unreliability of ampicillin monotherapy against *E. coli* while illustrating generally favorable susceptibility to aminoglycosides and carbapenems.

Non-*E. coli* Gram-negative organisms accounted for a higher proportion of UTI below 1 month of age and in infants with healthcare exposure or urinary tract anomalies, a pattern consistent with the broader neonatal infection literature. In very preterm infants managed in the neonatal intensive care unit (NICU), *Candida* species may predominate and must be considered when bacteriological cultures are negative in an infant who fails to respond to antibacterial therapy. Contemporary uropathogen distribution data specific to UTI in the 0–90-day age band, stratified by age and acquisition setting, are not available from multi-center population-based sources; thus, local surveillance data are essential. Table 1 summarizes the available uropathogen and resistance data relevant to empiric antibiotic decision-making.

### 3.2. Resistance Patterns

Ampicillin resistance among *E. coli* uropathogens is common. In a retrospective review of 687 infants younger than 6 months with community-acquired *E. coli* UTI, in vitro ampicillin susceptibility was 45.2% in the non-ESBL group and 0% in the ESBL group [12]. This limits ampicillin monotherapy as an empiric agent for *E. coli*, although ampicillin remains essential for Enterococcal and Group B Streptococcal coverage.

Extended-spectrum beta-lactamase (ESBL)-producing *E. coli* accounted for 15.3% of community-acquired *E. coli* UTI isolates in infants younger than 6 months in a Korean tertiary center cohort [12]. ESBL prevalence varied widely by country and setting, from under 5% in some European community series to over 90% in healthcare-associated settings in low- and middle-income countries [13]; the latter figure reflects a different epidemiological context and should not be applied to community-acquired UTI in high-income settings. ESBL-producing *E. coli* typically produces CTX-M-type enzymes and are resistant to all standard cephalosporins, while retaining susceptibility to amikacin (100%), piperacillin–tazobactam (95.8%), and, in approximately two-thirds of cases, to amoxicillin–clavulanate (63.8%) [12]. These susceptibility patterns directly inform definitive therapy for confirmed ESBL UTI (Section 5.3).

Healthcare-associated infection, prior antibiotic exposure, prematurity, and NICU admission are the principal risk factors for UTI caused by multidrug-resistant Gram-negative organisms, including ESBL-producing and, in high-burden settings, carbapenem-resistant organisms. Empiric antibiotic selection must account for these factors, as discussed in Section 4.3.

## 4. Initial Empiric Antibiotic Selection

Empiric antibiotic therapy must be initiated before culture and susceptibility results are available, targeting the most likely uropathogens while accounting for local resistance rates and the clinical context of each infant. In neonates and young infants, empiric selection is shaped by four considerations: (1) the uropathogen spectrum (Section 3); (2) the need for Gram-positive coverage in the youngest infants; (3) local resistance prevalence; and (4) antibiotic safety constraints specific to this age group, particularly the neonatal ceftriaxone caution described below. No randomized trial has directly compared empiric antibiotic regimens in confirmed UTI restricted to infants aged 0–90 days.

### 4.1. Regimen Selection for Community-Acquired UTI in Term Neonates and Young Infants

A combination of a parenteral aminopenicillin (ampicillin or amoxicillin) plus an aminoglycoside (gentamicin) has been the most widely used empiric regimen for neonates with community-acquired UTI in European countries. This combination provides broad Gram-negative as well as Enterococcal and Group B Streptococcal coverage, and synergistic bactericidal activity [14,15]. The pharmacokinetic rationale for amoxicillin in neonates is supported by a pooled population pharmacokinetic analysis demonstrating oral bioavailability of 87% and adequate target attainment across gestational and postnatal ages when given twice daily in the first weeks of life [16]. Clinical data in term neonates receiving oral amoxicillin confirmed that therapeutic serum concentrations are achievable [17].

A third-generation cephalosporin (cefotaxime or ceftazidime) is an alternative for Gram-negative coverage; cefotaxime was used empirically in 25.7% of ESBL and 28.2% of non-ESBL *E. coli* UTI cases in a cohort of infants younger than 6 months [12], and initial oral antibiotic treatment was used in 63% of infants under 1 year of age in a Swedish national study of 1306 infants, predominantly third-generation cephalosporins [18]. No head-to-head comparison of ampicillin plus gentamicin versus a third-generation cephalosporin exists for UTI in 0–90-day-old infants. An indirect comparison from a systematic review and meta-analysis commissioned by the World Health Organization (WHO), of four randomized trials in young infants aged 0–59 days with sepsis or possible serious bacterial infection, found no significant difference in treatment success between parenteral third-generation cephalosporins and ampicillin or penicillin plus aminoglycoside (RR 1.03, 95% CI 0.93–1.13; n = 1083; moderate certainty of evidence) [19]. Notably, cephalosporins were associated with a lower rate of adverse events including jaundice compared with ampicillin or penicillin plus aminoglycoside (RR 0.35, 95% CI 0.15–0.82; moderate certainty) [19], though this was in a sepsis/possible serious bacterial infection population and does not negate the specific neonatal risks of ceftriaxone described below. The confidence interval for this comparison (RR 0.93–1.13) does not exclude a clinically meaningful difference in treatment success, and the estimate derives from only four trials with limited enrollment of infants ≤ 90 days with confirmed UTI; thus, it should be applied as indirect supporting evidence only.

### 4.2. Ceftriaxone: Neonatal Safety Considerations

Ceftriaxone should be avoided as an empiric or definitive agent in neonates and used with caution in young infants in this age group. Two distinct safety concerns are established.

○ Bilirubin displacement: Ceftriaxone competes with bilirubin for albumin binding sites, raising the risk of bilirubin encephalopathy in jaundiced neonates. A systematic review of nine studies identified increases in serum bilirubin necessitating antibiotic change in a subset of neonates and biliary sludge in 6 of 80 infants, self-resolving in all cases [20]. The clinical relevance of bilirubin displacement appears greatest in infants with pre-existing hyperbilirubinemia.
○ Calcium interaction: Concomitant administration of ceftriaxone and calcium-containing intravenous solutions has been associated with fatal cardiopulmonary events in neonates. A systematic review identified eight cardiopulmonary adverse events attributable to this interaction, including seven neonatal deaths [20]. The United States Food and Drug Administration (US FDA) issued an alert in 2007 contraindicating co-administration within 48 h in patients of any age; a 2009 modification permitted sequential use in patients older than 28 days, provided intravenous lines are thoroughly flushed [20].

The authors of the systematic review noted that comparable alternative agents are available and that the risks of ceftriaxone use in neonates may outweigh the benefits [20]. Cefotaxime is therefore the preferred third-generation cephalosporin when a parenteral cephalosporin is indicated in the neonatal period, as it does not share the bilirubin-displacement or calcium-interaction concerns of ceftriaxone. The American Academy of Pediatrics similarly recommends using alternative therapy when ceftriaxone is co-administered with any IV calcium-containing solution, including parenteral nutrition, and advises caution in hyperbilirubinemic neonates, particularly those born preterm [21]. This restriction applies most stringently to neonates aged 0–28 days, for whom all intravenous calcium-containing solutions (including parenteral nutrition) and hyperbilirubinemia represent absolute contraindications to ceftriaxone usage. In young infants older than 28 days without jaundice and not receiving calcium-containing infusions, ceftriaxone may be used, provided intravenous lines are thoroughly flushed between infusions where sequential administration is unavoidable [20,21].

### 4.3. Age-Specific Considerations

All neonates (age 0–28 days) with confirmed or strongly suspected UTI should be hospitalized and treated with initial parenteral antibiotics. Neonates are classified to be in a complicated-UTI subgroup in the European Society for Pediatric Infectious Diseases (ESPID) guideline, warranting initial IV therapy with consideration of longer treatment duration [22]. Empiric cover must include *E. coli* and other Gram-negative organisms, *Enterococcus faecalis*, and Group B *Streptococcus*; ampicillin plus gentamicin would meet these requirements where local susceptibility permits. Cefotaxime-based regimens are an alternative but do not provide Enterococcal coverage.

Young infants (age 29–90 days) with confirmed or suspected UTI and a systemic sepsis presentation, hemodynamic instability, or age below 60 days are generally admitted and treated parenterally [22]. Well-appearing young infants beyond 60 days with a clear lower-tract UTI focus, and low risk of concurrent meningitis may be candidates for initial or early oral therapy in some practice contexts, although the evidence base for this in culture-confirmed UTI remains limited (Section 6). Ceftriaxone is appropriate in young infants older than 28 days as mentioned above [20,21].

### 4.4. Healthcare-Associated and NICU-Acquired UTI

Healthcare-associated UTI, those occurring in hospitalized infants with prolonged NICU stays, prior antibiotic exposure, invasive procedures, or indwelling urinary catheters, carries a substantially higher risk of resistant Gram-negative infection, including ESBL-producing *Klebsiella pneumoniae* and *Pseudomonas aeruginosa*. Empiric coverage should be broadened beyond the standard community-onset regimen, guided by unit-specific surveillance data and the infant’s known colonization status. An antipseudomonal beta-lactam combined with an aminoglycoside would be appropriate for empiric therapy when *Pseudomonas* or highly resistant Gram-negative organisms are plausible. UTI-specific uropathogen and treatment outcome data for healthcare-associated UTI in infants 0–90 days of age are not available from the current literature.

### 4.5. Antibiotic Dosing in Neonates and Young Infants

Antibiotic dosing in this age group requires adjustment for gestational age (GA) and postnatal age (PNA), which together determine the maturation of renal clearance. Population pharmacokinetic modeling in 261 preterm and term neonates demonstrated substantial increases in amoxicillin clearance with advancing postnatal age, reflecting glomerular filtration rate maturation [16]. GA and PNA must therefore be considered when selecting dose and interval for any antibiotic throughout the neonatal period. Specific model-derived oral and intravenous amoxicillin dosing recommendations by GA and PNA have been provided [16]. Clinicians should note that the Reduction of Intravenous Antibiotics In Neonates (RAIN) trial administered amoxicillin–clavulanate every 8 h [23], while the twice-daily schedule was derived from the Keij 2023 pharmacokinetic model of Keij et al. [16], published after the RAIN trial. Both schedules have been used clinically; the twice-daily regimen is pharmacokinetically preferred for the first week of life. Gentamicin dosing in neonates requires individualized interval selection based on gestational and postnatal age, with intervals typically ranging from every 24 to every 48 h reflecting renal maturation; therapeutic drug monitoring is essential to ensure optimal dosing [21].

## 5. Pathogen-Directed Therapy: Narrowing After Susceptibility Results

Once culture and susceptibility results are available, typically 24–48 h after urine specimen collection, empiric therapy should be reviewed and narrowed to the most targeted effective agent. Prompt de-escalation limits unnecessary antibiotic pressure, reduces the selection of resistant organisms, and is the central principle of antimicrobial stewardship in this context. De-escalation decisions must account for the concurrent bacteremia and meningitis workup, which may influence the choice and route of administration of the antibiotics.

### 5.1. Susceptible E. coli and Other Gram-Negative Organisms

For susceptible *E. coli*, therapy should be narrowed from the empiric combination therapy to a single targeted antibiotic. Amoxicillin is appropriate for susceptible isolates. Third-generation cephalosporins (intravenous cefotaxime; oral cefixime) are alternatives. The narrowest effective agent should be selected. Fluoroquinolones should not be used in this age group because of toxicity concerns during the developmental age [21]. Trimethoprim-sulfamethoxazole is contraindicated in infants under 8 weeks of age due to the risk of kernicterus [21], and its susceptibility is low against ESBL-producing isolates (41% versus 73.8% in non-ESBL *E. coli*) [12].

Nitrofurantoin is also contraindicated in infants younger than 1 month due to the risk of hemolytic anemia in infants with immature erythrocyte enzyme systems [21]; it should not be used as a step-down oral agent in neonates or young infants younger than 1 month.

### 5.2. Enterococcus and Group B Streptococcus

Ampicillin or amoxicillin is the definitive agent of choice for *Enterococcus faecalis*, which has a lower minimum inhibitory concentration for ampicillin than for any other beta-lactam [12]. Aminoglycosides should be continued for synergy in bacteremic disease, as they are not effective as monotherapy against *Enterococcus*. For Group B *Streptococcus*, penicillin or ampicillin is the definitive agent. Suspicion of *Staphylococcus* aureus UTI in this age group should prompt imaging to exclude an underlying urinary tract anomaly.

### 5.3. ESBL-Producing Enterobacterales

ESBL-producing *E. coli* are considered resistant to all cephalosporins and to ampicillin as monotherapy. The following options were evaluated in a retrospective review of 105 infants younger than 6 months with community-acquired ESBL *E. coli* UTI, all treated with non-carbapenem antimicrobials [12]:

○ Carbapenems (imipenem-cilastatin or meropenem) showed 100% in vitro susceptibility and could be appropriate for severe or bacteremic ESBL infection; carbapenem use should be reserved for cases where non-carbapenem alternatives are not suitable, to preserve this drug class.
○ Piperacillin–tazobactam had a susceptibility of 95.8% in the ESBL group and could be a viable non-carbapenem option for ESBL-associated UTI.
○ Amoxicillin–clavulanate showed susceptibility of 63.8% in the ESBL group. This could be an appropriate non-carbapenem agent in susceptible isolates for step-down to oral therapy, noting that the 4:1 amoxicillin/clavulanate ratio is preferred in neonates to ensure adequate clavulanate exposure [24]. Ratios higher than 4:1, including the 7:1 formulation, provide less clavulanate per dose and have been shown to result in a lower probability of achieving the pharmacodynamic target in neonates; the 4:1 formulation is therefore the appropriate choice for step-down therapy in this age group [24]. High urinary concentrations may contribute to clinical success even with borderline in vitro susceptibility.
○ Amikacin with a susceptibility of 100% in the ESBL group may be an important carbapenem-sparing option for parenteral therapy where oral antibiotic step-down is not appropriate. Therapeutic drug monitoring is required for appropriate dosing.

In a 2021 study, no significant differences in defervescence time (1.2 days in both groups), relapse rate (3.8% ESBL versus 2.6% non-ESBL, *p* = 0.513), or clinical cure were observed between the ESBL and non-ESBL groups of patients treated with non-carbapenem antimicrobials [12]. Critically, no patient in the ESBL group had concomitant bacteremia, so these results do not establish the adequacy of non-carbapenem therapy for ESBL UTI with bloodstream infection. It should be noted that this cohort had a mean age of approximately 2.8 months; data specific to infants aged 0–90 days were not separately reported, and findings reflect a single-center Korean population; local susceptibility patterns should therefore guide clinical decisions. For ESBL-UTI with bacteremia, a carbapenem is preferred until a clinical and microbiological response is confirmed, with de-escalation thereafter if the susceptibility profile allows.

### 5.4. AmpC-Producing and Carbapenem-Resistant Organisms

AmpC-producing Enterobacterales (*Enterobacter* spp., *Serratia*, *Citrobacter*, *Morganella*) may exhibit inducible resistance to third-generation cephalosporins despite apparent initial susceptibility. Therefore, these agents should not be used as definitive therapy for AmpC-producing organisms, even when the initial susceptibility report appears favorable [22]. Cefepime or a carbapenem is preferred for definitive therapy of AmpC-producing isolates where clinical severity warrants it [22]. No age-specific UTI treatment data for AmpC infection in infants aged 0–90 days exist in the published literature. Carbapenem-resistant organisms require specialist infectious disease input to guide treatment. Both represent declared gaps in evidence for this population (Section 12).

## 6. Route of Therapy

The choice between intravenous and oral antibiotic therapy is the central treatment decision in neonatal and young-infant UTI and represents the highest-yield antimicrobial stewardship opportunity in this population. The following sections address the evidence for initial parenteral therapy, IV-to-oral transition, oral antibiotic pharmacokinetics, and the criteria for transition to safe step-down therapy.

### 6.1. Intravenous Therapy: Initial Route and Duration

Initial intravenous therapy is the standard of care for UTI in neonates (age 0–28 days) and for young infants with features suggesting complicated infection, bacteremia, or clinical instability. This practice reflects the risk profile of the age group and is endorsed by guideline consensus [22], rather than by comparative trials of initial route in confirmed UTI.

Wide variation in the duration of intravenous therapy for neonatal UTI has been documented. A physician-preference survey using a 2-week-old neonatal UTI vignette found that general pediatricians and hospitalists preferred a median duration of 2 days, pediatric infectious disease specialists preferred 5 days, and neonatologists preferred 7 days [25]. This variation is not supported by outcome differences and identifies IV duration as a modifiable stewardship target.

The most comprehensive evidence on intravenous duration comes from a systematic review of 18 studies totaling 16,615 infants aged ≤ 90 days [5]. For nonbacteremic UTI, the largest component study (n = 3973 infants ≤ 60 days, 46 children’s hospitals, 2005–2015) found readmission for UTI in 1.5% of infants regardless of whether they received short (≤3 days) or long (≥4 days) IV therapy (aOR for long course 0.93, 95% CI 0.52–1.67), and documented a dramatic secular decline in long IV courses from 50% in 2005 to 19% in 2015 without any increase in readmissions [26]. Crucially, age-stratified analysis showed no significant difference in UTI readmission between short and long IV courses even in infants < 15 days of age (2.6% vs. 2.3%) [26]. A second large component study (n = 12,333 infants < 6 months) likewise showed no association between IV duration and treatment failure in 12,333 infants < 6 months [27]. For bacteremic UTI, ≤7 days of IV antibiotics showed no significant difference in recurrence compared with longer courses; the 30-day UTI recurrence rate was low across the two largest studies (5% and 2%, respectively) [5]. Across all studies in the systematic review, 67–100% of recurrences occurred in infants with vesicoureteral reflux, suggesting that an underlying anatomical anomaly, rather than treatment duration, may be the primary driver of recurrence risk [5]. These findings should be interpreted in the context of the available evidence: all 18 studies were observational, the two included RCTs enrolled few infants ≤ 90 days old, and 11 of 18 studies reported only IV duration without data on total antibiotic course length.

In term neonates aged ≤ 28 days specifically, a retrospective chart review of 112 neonates at two academic pediatric hospitals found a median IV duration of only 49 h (IQR 40–72 h), with 49% receiving ≤48 h. Only 1/112 (0.9%) experienced treatment failure. The predictors of longer IV courses were not clinical severity but administrative factors: subspecialty consultation (aOR 4.79, 95% CI 1.87–12.3), additional indication extending hospitalization (aOR 3.2, 1.2–8.7), and abnormal renal ultrasound (aOR 2.26, 1.01–5.08); neonates < 7 days of age also received longer courses [10]. These data, in term neonates without bacteremia or known genitourinary (GU) anomalies, support that short IV courses are already widely practiced in this age group and that decisions to prolong them are driven by clinical complexity rather than evidence of benefit.

Among infants with nonbacteremic UTI, 167/403 (41%) received short-course IV (≤3 days); treatment failure occurred in only 8/167 (5%) infants in the short-course group, with no significant association between IV duration and treatment failure on multivariate analysis (adjusted OR for long versus short course 0.42, 95% CI 0.11–1.63). Predictors of treatment failure were fever on admission (OR 3.37), prior UTI history (OR 6.87), and length of stay >15 days (OR 6.56). The rate of renal scarring on dimercaptosuccinic acid (DMSA scan was significantly higher in those who received long-course IV antibiotics (*p* = 0.03) [9], the only direct evidence in this age group linking IV duration to a measurable long-term adverse outcome. This association should be interpreted with caution. About 93% of bacteremic infants were in the long-course group, representing an important source of severity confounding, as bacteremia and underlying urinary tract anomaly may independently predispose these infants to renal scarring regardless of IV duration.

A second single-center cohort of 451 infants ≤ 90 days confirmed that among 427 infants with nonbacteremic UTI, treatment with <48 h of IV antibiotics resulted in failure in only 1/257 (0.4%) infants; no serious complications occurred in the short-course group; and all six total treatment failures were associated with prematurity, urinary tract anomaly, or antibiotic-resistant organisms rather than IV course length [11]. Among 10 bacteremic infants treated with <7 days of IV antibiotics, no treatment failure or serious complication occurred [11].

A meta-analysis in infants < 2 months confirmed a pooled adjusted OR for 30-day UTI recurrence of 1.02 (95% CI 0.64–1.61; *p* = 0.95; n = 5451; I^2^ = 0%; GRADE LOW) for ≤3 versus >3 days of parenteral antibiotics, with shorter courses associated with a mean reduction in length of stay of 3.5 days (95% CI 5.0–1.9). Subgroup analyses showed no significant difference in recurrence according to the duration of parenteral treatment in neonates ≤ 1 month (recurrence 2.2%, 95% CI 1.7–2.9%) or in infants 1–2 months (recurrence 1.5%, 95% CI 1.1–2.1%), or by bacteremia status [6]. These consistent findings across two systematic reviews and multiple cohorts support that prolonged IV courses in stable infants with UTI do not reduce recurrence and extend hospitalization without clinical benefit.

### 6.2. Intravenous-to-Oral Antibiotic Step-Down

Early IV-to-oral transition is supported by direct UTI evidence and by indirect evidence from neonatal infection trials, and should be the standard approach for infants meeting the transition criteria described in Section 6.3.

#### 6.2.1. Direct UTI Evidence

In a multicenter retrospective cohort of 115 infants aged ≤ 60 days with bacteremic UTI across 11 children’s hospitals, a short parenteral course (≤7 days, 50% of the cohort) was not associated with higher 30-day UTI recurrence compared with longer courses (adjusted risk difference 3%, 95% CI −5.8 to 12.7). Same-organism recurrence did not differ (adjusted risk difference 0.2%, 95% CI −7.8 to 8.3). Length of stay was significantly shorter in the short-course group (adjusted mean difference 6 days, 95% CI 4.0–8.8). The authors concluded that short parenteral therapy with early oral conversion may be considered in this population [8]. These findings should be interpreted with caution, as the study enrolled infants aged ≤ 60 days rather than the full 0–90 day age range of this review, and was not powered to detect small but potentially clinically meaningful differences in recurrence rates.

#### 6.2.2. Indirect Evidence from Non-UTI Neonatal Infection Trials

The RAIN trial was the first randomized controlled trial of IV-to-oral antibiotic switch in neonates conducted in a high-income setting. In this multicenter, open-label, non-inferiority trial across 17 Dutch hospitals, 510 neonates (postmenstrual age ≥ 35 weeks, postnatal age 0–28 days, weight ≥ 2 kg) with probable bacterial infection were randomized after 48 h of IV therapy to oral amoxicillin–clavulanate (75/18.75 mg/kg/day in three divided doses, 4:1 ratio) or continued IV antibiotics for a total 7-day course. Non-inferiority of the oral switch strategy for 28-day cumulative bacterial reinfection was demonstrated. Hospitalization duration and IV catheter reinsertions were significantly reduced. No increase in adverse events or study-related mortality was observed [23].

Three critical features of RAIN limit its direct applicability to UTI management. First, the enrolled population had probable bacterial infection with sterile blood cultures: proven bloodstream infection was an explicit exclusion criterion, and the trial protocol states that results ‘cannot directly be applied to culture-proven infection.’ Second, proven bacteremia was excluded. RAIN does not address oral step-down in bacteremic UTI. Third, premature neonates below 35 weeks postmenstrual age and infants weighing less than 2 kg were excluded. RAIN is therefore cited as indirect supporting evidence for oral step-down in stable neonates, not as a UTI-specific trial.

Keij and colleagues also published a systematic review and meta-analysis of oral antibiotic use in neonates (0–28 days) encompassing 31 studies. Pooled analyses showed equal relapse rates (OR 0.95, 95% CI 0.79–1.16; I^2^ = 0%) and comparable mortality (OR 1.11, 95% CI 0.72–1.72; I^2^ = 0%) between oral and IV therapy, with a reduction in hospital stay observed in studies reporting this outcome [14]. The review confirmed that adequate serum concentrations are achievable following oral antibiotic administration in neonates but noted that robust UTI-specific efficacy data were lacking.

### 6.3. Oral Antibiotic Selection and Dosing

When oral step-down is indicated, amoxicillin–clavulanate has been the most extensively studied oral agent in neonates and was the agent used in the RAIN trial. A pooled population pharmacokinetic study in 261 preterm and term neonates confirmed an oral amoxicillin bioavailability of 87%, comparable to that in adults, though with slower absorption [16]. A twice-daily oral regimen achieved superior target attainment compared with three- or four-times daily dosing in the first week of life, attributable to the delayed absorption profile of oral amoxicillin in neonates. Specific model-derived oral and intravenous amoxicillin dosing recommendations by GA and PNA are reported [16].

Oral clavulanic acid bioavailability was substantially lower than amoxicillin: 24.4% in neonates up to 10 days of age [24]. A companion population pharmacokinetic analysis demonstrated that the 4:1 amoxicillin/clavulanate ratio achieved better clavulanate target attainment than higher amoxicillin ratios (7:1 or 16:1) across the full probability-of-target-attainment range. Formulations with ratios above 4:1 may result in inadequate clavulanate exposure in neonates [24].

Therapeutic oral amoxicillin concentrations above target were confirmed in a clinical study of 222 term neonates with early-onset group B Streptococcal infection who were switched from intravenous to oral amoxicillin after 48 h; none had trough levels below the therapeutic threshold [17]. For young infants beyond the neonatal period (age 29–90 days), antibiotic selection generally included a third-generation oral cephalosporin (e.g., cefixime) or amoxicillin–clavulanate used according to susceptibility. Antibiotic-specific outcome data in confirmed UTI restricted to the 29–90-day age band are not available from the current evidence base.

### 6.4. Criteria for Transition to Safe Oral Antibiotic

No prospectively validated clinical decision rule for oral step-down exists specifically for this age group. The criteria in Table 2 are synthesized from the eligibility criteria of trials demonstrating safe oral therapy, the explicit prerequisites stated in the neonatal oral switch literature, and standard principles of IV-to-oral transition applied to the relevant age. They should be regarded as *expert practice pending prospective validation* in this population. Each criterion should be documented before transition.

## 7. Duration of Antibiotic Therapy

Total antibiotic duration should be individualized based on the presence of bacteremia, the infecting organism, the presence of structural urinary abnormality, the clinical response, and meningitis status. Table 3 summarizes the recommended approach by clinical scenario.

### 7.1. Nonbacteremic UTI

For culture-confirmed nonbacteremic UTI in otherwise stable infants, a total antibiotic course of 7–10 days is consistent with pediatric guideline recommendations [22]. No randomized trial has directly compared total course lengths in infants aged 0–90 days. As detailed in Section 6.1, the evidence consistently demonstrates that the IV component can be shortened to ≤3 days without increased recurrence [5,6,11,12,27], supporting early transition to oral therapy to complete the total course.

The only meta-analytic evidence addressing total duration of antibiotic course in this age group found no significant difference in 30-day recurrence between ≤10 and >10 total days of therapy (pooled OR 1.29; 95% CI 0.45–3.66; *p* = 0.63; 5 observational studies; n = 491) [6]. Direct neonatal data from retrospective chart review of 112 term neonates aged ≤ 28 days with nonbacteremic UTI (bacteremia and GU anomalies excluded) found a median total antibiotic course of 10 days (IQR 10–14 days), with only 1/112 (0.9%) experiencing treatment failure within 30 days [10]. These data, from the population most directly relevant to neonatal UTI management, support a 10-day total course as an appropriate target for uncomplicated nonbacteremic UTI in term neonates.

### 7.2. Bacteremic UTI

Bacteremic UTI has historically prompted prolonged intravenous therapy, with courses of 10–14 days or longer as conventional practice. The available evidence does not support this approach in infants who respond to therapy and in whom meningitis has been excluded.

In the largest available study of bacteremic UTI in 115 infants aged ≤ 60 days across 11 children’s hospitals, infants receiving ≤7 days of parenteral antibiotics did not experience significantly higher 30-day UTI recurrence (adjusted risk difference 3%, 95% CI −5.8 to 12.7) or all-cause reutilization (adjusted risk difference 3%, 95% CI −14.5 to 20.4) compared with longer courses. Length of stay was substantially shorter in the short-course group (4.5 versus 10.8 days, adjusted mean difference 6 days, 95% CI 4.0–8.8). Thirteen infants in the long-course group were discharged with a peripherally inserted central catheter; one experienced a catheter-related complication. Eight infants (7%) had bacteremia lasting more than one day and received longer IV courses [8].

Consistent findings across multiple observational cohorts support this conclusion. In the largest multicenter study of bacteremic UTI in infants < 3 months of age (n = 251, 11 US institutions), the duration of parenteral antibiotics did not differ significantly between infants with and without 30-day recurrence (8.2 vs. 7.8 days, *p* = 0.81). Crucially, no infant had a relapsed bacteraemic UTI (0/251), and 6/251 (2.4%) had a relapsed UTI (without bacteremia); all relapses were associated with vesicoureteral reflux on voiding cystourethrography, confirming that underlying anatomy, not treatment duration, drove recurrence risk [28]. In the Melbourne single-center cohort, none of the 10 bacteremic infants aged 90 days treated with <7 days of IV antibiotics experienced treatment failure or serious complications [11]. Additionally, in the Toronto cohort of 403 infants aged < 60 days, the rate of renal scarring on DMSA scan was significantly higher in those who received long-course intravenous antibiotics (*p* = 0.03) [9]; this association was subject to severity confounding, as bacteremic infants preferentially received longer courses and may be predisposed to renal scarring independent of IV treatment duration. These consistent findings support a total course of 10–14 days, IV followed by oral, as an evidence-consistent approach to managing bacteremic UTI in infants aged ≤ 90 days.

### 7.3. Meningitis: Exclusion and Its Implications

Concomitant bacterial meningitis with UTI fundamentally alters both route and duration of antibiotic therapy: intravenous treatment must be continued at CNS-adequate doses for the full meningitis course (typically 14–21 days depending on organism), and oral step-down would be inappropriate [22]. Clarification of meningitis status is therefore a prerequisite for any decision to shorten IV therapy or to initiate a transition to an oral antibiotic.

In a systematic review and meta-analysis of 20 cohort studies of infants aged 29–90 days with UTI who underwent lumbar puncture, pooled concomitant bacterial meningitis prevalence was 0.25% (95% CI 0.09–0.70%) [29]. This low prevalence supports a selective approach to lumbar puncture in this age band and underpins the recommendation to proceed with short IV courses and oral step-down once meningitis has been excluded. Consistent with this, no case of meningitis was identified in febrile infants over 60 days of age or in those with confirmed UTI [4].

A larger and more recent meta-analysis of 39 studies confirmed a similar overall pooled prevalence of 0.3% (95% CI 0.1–0.4%; I^2^ = 0%) [30]. Importantly, age-stratified analysis found a higher prevalence in neonates ≤ 28 days (0.7%, 95% CI 0.3–1.1%) compared with infants aged 29–90 days (0.2%, 95% CI 0.0–0.3%); the within-study risk difference between these two age bands was not statistically significant, supporting a selective rather than universal approach to lumbar puncture across the full age range, with a lower threshold retained for neonates ≤ 28 days pending further age-specific data [30].

For neonates aged 0–28 days, concomitant meningitis data are extremely limited. Both studies cited above had small-sample in this age band [4,29,30], and the recommendation for CSF evaluation before shortening IV therapy or initiating oral antibiotic transition is therefore more conservative in neonates than in older infants [22]. Where lumbar puncture is deferred, declined, or non-diagnostic, empiric CNS-adequate antibiotic dosing should be maintained until CSF status is clarified [22].

## 8. Urinary Tract Abnormalities and Complicated Urinary Tract Infection

Congenital anomalies of the kidney and urinary tract (CAKUT) define a complicated-UTI phenotype in which standard management assumptions require modification. Neonates are classified in a complicated-UTI subgroup in the ESPID guideline, warranting initial intravenous antibiotics and consideration of longer treatment duration [22]. *Staphylococcus aureus* bacteremia in the context of UTI should raise suspicion of an underlying urinary tract anomaly [8]. A case of neonatal obstructive uropathy illustrates that in structurally severe cases, surgical source control is the key intervention and antibiotics alone are insufficient [31].

Treatment practices for complicated UTI in children vary substantially. In a multicenter retrospective cohort of 510 children aged 0–17 years with urinary tract anomalies and UTI, significant variation was observed between hospitals in IV antibiotic spectrum, IV treatment duration, and hospital length of stay. Despite this variability, 30-day emergency department return rates, ICU admissions, and bacteremia rates did not differ across hospitals [32], suggesting that much of the observed practice variation may have represented overuse of antibiotics rather than clinical necessity.

For infants with confirmed or suspected urinary tract anomaly and UTI, the following modifications to standard treatment are recommended. Empiric antibiotic coverage should be broader, including anti-Gram-positive organisms (ampicillin) and antipseudomonal agents where structurally complicated or healthcare-associated infection is plausible [22]. Oral step-down should be deferred until structural diagnosis is clarified and source-control measures are in place or excluded [22]. A total antibiotic course of at least 10–14 days is appropriate for complicated presentations [22]. Urological assessment should be sought early when obstruction is suspected [22]. It should be noted that all recommendations in this section represent expert practice, as no treatment-specific evidence exists for infants aged 0–90 days in the current literature.

## 9. Treatment Failure and Persistent Bacteremia

Treatment failure is defined as persistent fever, clinical deterioration, positive follow-up culture, or early recurrence. It should prompt a structured reassessment rather than empiric antibiotic escalation [22]. No study has specifically examined the management of treatment failure or persistent bacteremia in neonates and young infants with UTI. The following approach is derived from clinical principle and extrapolation from broader evidence. When an infant fails to improve or has persistently positive blood cultures, the following questions should be addressed:

○ Source control: Renal and bladder ultrasound imaging is indicated. Antibiotic therapy alone will not sterilize an obstructed infected system.
○ Organism and susceptibility: Repeat cultures from blood and urine should be obtained. It would be important to confirm that ESBL, AmpC, or resistant organisms have not been missed.
○ Adequacy of the current regimen: Is the agent appropriate for the confirmed organism? Is the dose correct for the infant’s current gestational and postnatal age?
○ Oral absorption: Vomiting, diarrhea, or feeding difficulties may impair amoxicillin absorption, particularly in the first weeks of life.
○ Meningitis: If not previously excluded, cerebrospinal fluid evaluation should be considered in a neonate with persistently positive blood cultures.
○ Persistent bacteremia: Bacteremia persisting beyond 72 h of appropriate therapy warrants return to or continuation of IV therapy, repeat imaging, and infectious disease consultation.

Bacteremia lasting more than one day occurred in 7% of infants and was observed exclusively in the long-course treatment group [8], consistent with the practice of extending therapy when bacteremia is slow to clear. This is clinically reasonable but is not validated as a treatment strategy for persistent bacteremia in this age group specifically.

## 10. Antimicrobial Stewardship

The management of UTI in neonates and young infants offers several high-yield stewardship opportunities centered on avoiding unnecessarily prolonged intravenous therapy, facilitating timely transition to oral antibiotics, and restricting the use of broad-spectrum and carbapenem-class antibiotics. This section addresses stewardship opportunities specific to treatment decisions about antibiotic selection, route, and duration for UTI occurring in patients aged 0–90-days. For broader program-level stewardship design, readers are directed to established stewardship frameworks.

### 10.1. Reducing Unnecessarily Long Duration of Intravenous Antibiotics

A physician survey found marked variation in preferred IV duration across specialties when presented with the same neonatal UTI case scenario, a difference not supported by clinical outcome data [25]. A systematic review of 18 studies totaling 16,615 infants aged ≤ 90 days found 30-day UTI recurrence among 1.5–2.4% regardless of whether infants received shorter or longer IV courses, versus a 67–100% recurrence rate observed in those with vesicoureteral reflux [5]. A meta-analysis in infants < 2 months confirmed a pooled adjusted OR of 1.02 (95% CI 0.64–1.61; GRADE LOW) for recurrence with ≤3 versus >3 days of parenteral antibiotics, and shorter courses were associated with 3.5 fewer days of hospitalization [6]. Prolonged intravenous therapy adds risk of catheter-related complications, nosocomial infection, parent–child separation, and healthcare costs without improving outcomes in responding infants. A clinical pathway targeting a 3-day IV course in neonates with UTI demonstrated sustained reduction in IV antibiotic treatment duration and length of stay over 2 years with no readmissions [33].

### 10.2. Timely Transition from IV to Oral Antibiotic Step-Down

Early IV-to-oral switch in stable neonates with probable bacterial infection was shown to be non-inferior to a full IV course and significantly reduced hospitalization and catheter-related complications in the RAIN trial [23]. Oral amoxicillin achieved 87% bioavailability in neonates [16], and the 4:1 amoxicillin/clavulanate formulation was well tolerated in 99% of RAIN participants [24]. These findings support a structured step-down approach in eligible infants, with the eight transition criteria in Table 2 as the operational framework.

### 10.3. Susceptibility-Guided Narrowing and Carbapenem Restriction

Prompt de-escalation to the narrowest-spectrum antibiotic active against the confirmed uropathogen within 24–48 h of susceptibility results is the central stewardship principle for definitive therapy. For ESBL UTI without bacteremia, non-carbapenem therapy should be used preferentially to reserve the carbapenem class [12]. Carbapenem use should be reserved for bacteremic disease or clinical failure on non-carbapenem therapy.

### 10.4. Standardizing Practice

The significant variation between hospitals and between specialties in UTI management in this age group is a stewardship challenge in itself. Institutional pathways or clinical standards targeting IV antibiotic duration, oral antibiotic step-down eligibility, and total duration of antibiotic therapy can reduce overuse without worsening outcomes, as the neonatal UTI pathway study demonstrated [33]. The absence of national guideline recommendations specifically for neonatal UTI treatment has been cited as a barrier to such standardization [33], and developing this guidance is an urgent research and policy priority.

## 11. Practical Recommendations

The following recommendations synthesize the evidence reviewed in Section 3, Section 4, Section 5, Section 6, Section 7, Section 8, Section 9 and Section 10. Each is graded as evidence-supported (grounded in an observational study or indirect randomized controlled trial, with the population and indication caveat stated) or expert practice (derived from pharmacological reasoning, guideline consensus, or clinical principle where direct evidence was absent). No recommendation is stated more strongly than the evidence permits. Table 4 below provides a summary of these recommendations.

### 11.1. Special Situations

#### 11.1.1. Premature Infants (<37 Weeks Gestational Age)

No UTI-specific treatment evidence exists for premature infants [22]. Empiric regimens should be guided by the unit’s late-onset sepsis protocol, local uropathogen data, and known colonization status [22]. Dosing must account for gestational and postnatal age [16]. *Candida*-associated UTI must be considered in very preterm infants who fail to respond to antibacterial therapy [21]. Infectious disease consultation is strongly advised [22].

#### 11.1.2. Meningitis Not Yet Excluded

Where cerebrospinal fluid evaluation has not been performed or is non-diagnostic, empiric CNS-adequate antibiotic dosing should be maintained until the situation is clarified. Oral step-down and abbreviated IV courses are not appropriate until meningitis has been excluded, particularly in neonates aged ≤ 28 days [22,29].

#### 11.1.3. Treatment Failure

Non-response should prompt the systematic reassessment detailed in Section 9 (source control, organism and susceptibility, dosing adequacy, oral absorption, meningitis exclusion). Infectious disease consultation is recommended for persistent bacteremia, suspected resistant organism, or failure to respond within 48–72 h of apparently appropriate therapy [22].

## 12. Evidence Gaps and Research Priorities

The evidence gaps identified throughout this review are substantial. Urinary tract infection is a common, consequential infection in infants aged 0–90 days, yet—uniquely among common pediatric infectious syndromes—it has never been the subject of a randomized trial of antibiotic selection, route of administration, or treatment duration in this specific population. Table 5 organizes the gaps by priority.

The three highest-impact priorities are: (1) a randomized trial of oral antibiotic step-down versus full intravenous therapy in culture-confirmed UTI in neonates aged 0–28 days, extending the RAIN design to a UTI-specific, culture-proven population; (2) a prospective characterization of uropathogen distribution and resistance in UTI among 0–90-day by age band and acquisition setting; and (3) validated criteria for safe oral step-down in this age group. A recent narrative review on antibiotic duration in the neonatal intensive care unit, published in this journal, similarly identified the absence of randomized controlled trials of total antibiotic duration for UTI in the NICU setting as ‘one of the largest knowledge gaps in neonatal antimicrobial stewardship’ [35], underscoring the urgency of addressing these gaps.

## 13. Conclusions

Urinary tract infection in the first 90 days of life is a distinct and serious clinical problem that warrants management approaches tailored to this age group. The most clinically consequential decisions are: empiric antibiotic selection, duration of treatment with IV antibiotics, timing of transition to oral antibiotics, and management of bacteremic and resistant-organism infection. These decisions are often made in the near-total absence of evidence from randomized controlled trials performed specifically in this indication and age group.

Within this constraint, the available evidence supports the following positions. Initial intravenous therapy in neonates and ill-appearing young infants is appropriate and guideline-endorsed. Short intravenous courses, ≤3 days for nonbacteremic UTI and ≤7 days for bacteremic UTI, are not inferior in 30-day recurrence to longer courses in infants who are clinically responding, and reduce hospitalization without increasing adverse outcomes. IV-to-oral antibiotic step-down with amoxicillin–clavulanate is pharmacokinetically justified and clinically safe in term neonates ≥ 35 weeks postmenstrual age, provided meningitis is excluded, and the transition criteria are met. Non-carbapenem therapy is effective for ESBL-associated UTI without bacteremia in clinically improving infants younger than 6 months of age, with carbapenem reserved for bacteremic disease or clinical failure.

The strongest available randomized evidence for oral step-down, the RAIN trial, enrolled culture-negative neonates with probable bacterial infection and explicitly excluded proven bloodstream infection. It does not constitute a UTI-specific evidence base and must be presented as indirect supporting evidence whenever cited in this context. These limitations are not minor caveats; they are the current boundaries of the evidence, and all recommendations for oral therapy in confirmed neonatal UTI sit on the expert-practice side of those boundaries.

The persistent, wide variation in how neonatal UTI is managed reflects an unmet need that evidence synthesis alone cannot fully address. Intravenous antibiotic duration preferences ranging from 2 to 7 days across specialties for an identical clinical scenario underscore the urgent need for purpose-designed trials. The implementation of institution-specific clinical pathways or protocols for neonatal UTI and oversight of an antimicrobial stewardship team are practical and immediately actionable next steps toward standardizing therapeutic management in this age group. Well-designed clinical trials as specified in Section 12 are needed to develop strong evidence for improving health outcomes in this vulnerable population.

## Figures and Tables

**Table 1:** Uropathogens in neonates and young infants: approximate frequency, resistance concerns, and choice of empiric antibiotics.

Organism	Approximate Frequency *	Key Resistance Concern	Empiric Antibiotic Selection
*Escherichia coli*	~80% (bacteremic); 60% (all UTI < 60 d)	Ampicillin resistance common; rising ESBL	Primary empiric target; broaden coverage if local ESBL rates are high
*Klebsiella pneumoniae*	5–15% (higher in NICU)	ESBL; carbapenem resistance in low- and middle-income countries	Monitor; broaden cover if healthcare-associated
*Enterococcus faecalis*	3–13%	Ampicillin-susceptible; aminoglycoside-resistant	Ampicillin essential in combination regimens
*Staphylococcus aureus*	3–5% (associated with anomaly)	Methicillin resistance possible	*S. aureus* bacteremia + UTI: investigate for anomaly
Group B *Streptococcus*	2–4% (neonates)	Penicillin-susceptible	Ampicillin or penicillin adequate
*Enterobacter*/*Citrobacter*/*Serratia*	2–8% (NICU)	Inducible AmpC; 3rd-gen cephalosporin resistance	Avoid 3rd-gen cephalosporins for definitive therapy
*Pseudomonas aeruginosa*	Rare (community); NICU/anomaly	Intrinsic resistance; MDR emerging	Anti-pseudomonal cover if healthcare-associated
*Candida* spp.	Up to 54% in very preterm (NICU)	Antifungal, not antibiotic	Consider in very preterm not responding to antibiotics

* Frequencies are approximations derived from available studies, which differ by age, setting, and indication and are not directly comparable. *Candida* frequency is from a single very-preterm NICU cohort and should not be generalized. Local surveillance data take precedence over published estimates.

**Table 2:** Criteria for safe IV-to-oral antibiotic transition in neonates and young infants with urinary tract infection.

Criterion	Detail
1. Clinical improvement	Afebrile ≥ 24 h; improving inflammatory markers; no clinical deterioration
2. Hemodynamic stability	No vasopressor requirement; tolerating enteral feeds without vomiting
3. Bacteremia resolved or unlikely	Follow-up blood culture negative, or initial blood culture negative
4. Meningitis excluded	Cerebrospinal fluid culture negative or lumbar puncture results not indicating meningitis (Section 7.3)
5. Susceptible organism confirmed	Culture and susceptibility available; an effective oral agent exists
6. Oral absorption assured	Tolerating oral feeds without significant vomiting; no GI compromise
7. No uncontrolled structural source	No urinary obstruction or abscess requiring source control before switch
8. Caregiver capability confirmed	Reliable oral drug administration demonstrated before discharge

The RAIN trial discharge criterion—tolerating at least two oral doses and being clinically well—is the closest available validated implementation rule, but applies only to the culture-negative, non-UTI population enrolled in that trial.

**Table 3:** Recommended duration of IV and oral antibiotic therapy by clinical scenario in neonates and young infants with urinary tract infection.

Clinical Scenario	IV Duration	Oral Completion	Total Course	Evidence Basis
Nonbacteremic UTI, stable infant	≤3 days (≤48 h in selected cases)	Yes—when transition criteria met	7–10 days	Observational † [5,11,12,26,27]
Bacteremic UTI, meningitis excluded, clinical response	≤7 days	Yes—after clinical response	10–14 days	Observational † [8,11,28]
Bacteremic UTI, meningitis not yet excluded	Full IV; CNS-adequate dosing guided by CSF findings	Defer	14 days minimum	Expert practice ‡
Complicated UTI/urinary anomaly	Initial IV; duration by response and source control	Case by case	≥14 days	Expert practice ‡ [22]
Premature/NICU infant	No UTI-specific trial; follow clinical and PK guidance	Case by case	Case by case	Expert practice ‡

† Observational evidence only; no randomized trial has compared IV duration or total course length in culture-confirmed UTI in infants 0–90 days. ‡ Expert practice: recommendations based on pharmacological principles, ESPID guideline consensus [22], and clinical reasoning where direct trial evidence is absent; the authors take responsibility for these recommendations. CNS, central nervous system; IV, intravenous; NICU, neonatal intensive care unit; PK, pharmacokinetics.

**Table 4:** Summary of practical recommendations for antibiotic treatment of urinary tract infection in neonates and young infants aged 0–90 days.

Recommendation	Strength	Key Reference(s)
Hospitalize all neonates (age 0–28 d) and ill-appearing or high-risk young infants; initiate parenteral antibiotics.	Expert practice	[22]
Ampicillin (or amoxicillin) plus gentamicin is the preferred empiric regimen for term neonates with community-acquired UTI where local resistance permits.	Expert practice (PK support)	[14,15,16,17]
Cefotaxime is the preferred third-generation cephalosporin in neonates. Avoid ceftriaxone—bilirubin displacement risk and seven neonatal deaths reported from calcium interaction.	Expert practice (safety-based)	[21,22]
Broaden empiric coverage in healthcare-associated UTI, NICU, or known anomaly to cover resistant Gram-negatives.	Expert practice	[13,34]
Narrow to the most targeted effective agent within 24–48 h of susceptibility results.	Expert practice	[22]
For ESBL UTI without bacteremia, non-carbapenem therapy (piperacillin–tazobactam; amoxicillin–clavulanate where susceptible; amikacin) is reasonable in clinically improving infants.	Evidence-supported (observational, 0–6 months)	[12]
For ESBL UTI with bacteremia, a carbapenem is preferred until clinical and microbiological response is established.	Expert practice	[12]
Short parenteral courses (≤3 d in nonbacteremic; ≤7 d in bacteremic) are not inferior in 30-day recurrence to longer courses in responding infants.	Evidence-supported (observational, direct)	[5,10,11,12,27]
IV-to-oral step-down is appropriate when the eight transition criteria (Table 2) are met. Meningitis must be excluded before switching.	Evidence-supported (criteria synthesis)	[10,23,29]
Oral amoxicillin–clavulanate (4:1 ratio, twice daily) is the preferred oral step-down agent in neonates ≥ 35 weeks PMA and weighing ≥2 kg.	Evidence-supported (PK; indirect)	[16,24]
Total duration: 7–10 days for nonbacteremic UTI; 10–14 days for bacteremic UTI.	Expert practice	[22,29]
In infants aged 29–90 d, concomitant meningitis risk with UTI is low (0.25%); a selective LP approach is appropriate. Lower CSF-evaluation threshold is prudent in neonates ≤ 28 d.	Evidence-supported (29–90 d); expert practice (≤28 d)	[4,29]
Therapeutic drug monitoring for gentamicin is standard practice in neonates; dose and interval must be adjusted for gestational and postnatal age.	Expert practice	[21]

Evidence-supported = grounded in observational or indirect RCT evidence, with population/indication caveat stated. Expert practice = derived from pharmacological reasoning, guideline consensus, or clinical principle where direct evidence is lacking. ESBL, extended-spectrum beta-lactamase; IV, intravenous; LP, lumbar puncture; NICU, neonatal intensive care unit; PK, pharmacokinetics; PMA, postmenstrual age.

**Table 5:** Prioritized research gaps in antibiotic treatment of UTI in neonates and young infants aged 0–90 days.

Evidence Gap	Priority	Comment
Comparative empiric regimens in confirmed UTI among 0–90 d of age	High	No trial or comparative observational data. Ampicillin + gentamicin versus cephalosporin unanswered for this indication.
Uropathogen distribution specific to 0–90 d by age band and acquisition setting	High	All available data are either from broader age (<6 months, <1 year) or non-UTI indication.
Oral antibiotic step-down and oral-antibiotic only therapy in neonates ≤ 28 d with confirmed UTI	High	RAIN trial [23] excluded culture-proven infection and bacteremia. No UTI-specific RCT in this age group.
Total antibiotic course duration in infants aged 0–90 d	High	Existing evidence addresses IV component only. No comparison of total 7 vs. 10 vs. 14 days.
ESBL UTI with concomitant bacteremia in infants 0–90 d	High	No data with bacteremic ESBL cases [12]. Non-carbapenem adequacy in this combination is untested.
UTI treatment and dosing in premature/NICU infants	High	No UTI-specific treatment data. PK models extend to preterm for possible serious bacterial infection but not UTI.
Concomitant meningitis risk in neonates ≤ 28 d with UTI	High	Sample size too small in this age band [4,29].
AmpC-producing organism UTI in 0–90 d of age	Medium	No age-specific UTI evidence. Extrapolation from adult bacteremia data only.
Carbapenem-resistant UTI in 0–90 d of age	Medium	Essentially absent from the UTI-specific literature.
Validated oral step-down criteria	Medium	Section 6.4 criteria are synthesized; no prospective validation in age 0–90 d.
Stewardship intervention outcomes in 0–90 d UTI	High	Practice variability is documented; intervention-effect data are absent.
Management of treatment failure and persistent bacteremia	Medium	No management evidence in this age group; expert practice only.

High: directly limits current clinical decision-making. Medium: important but less immediately impactful on routine management.

## Data Availability

No new data were created or analyzed in this study. Data sharing is not applicable to this article.

## Abbreviations

CAKUT	Congenital anomalies of the kidney and urinary tract
CSF	Cerebrospinal fluid
DMSA	Dimercaptosuccinic acid
ESBL	Extended-spectrum beta-lactamase
ESPID	European Society for Paediatric Infectious Diseases
GA	Gestational age
IV	Intravenous
LP	Lumbar puncture
MDR	Multidrug-resistant
MIC	Minimum inhibitory concentration
NICU	Neonatal intensive care unit
PK	Pharmacokinetic
PMA	Postmenstrual age
PNA	Postnatal age
RAIN	Reduction of Intravenous Antibiotics In Neonates
UTI	Urinary tract infection
VUR	Vesicoureteral reflux
